# Supplementary material for: Detection of structural mosaicism from targeted and whole-genome sequencing data
Source: Genome Res. 2017 Oct;27(10):1704–14. doi: 10.1101/gr.212373.116 (PMC5630034; doi:10.1101/gr.212373.116)
Supplement: Supplemental Material [file supp_27_10_1704__index.html]

Detection of structural mosaicism from targeted and whole-genome sequencing data — Supplemental Material 

# Detection of structural mosaicism from targeted and whole-genome sequencing data

## Supplemental Material

- Supplemental\_Note\_S1.docx
- Supplemental\_Fig\_S1.pdf
- Supplemental\_Fig\_S2.pdf
- Supplemental\_Fig\_S3.pdf
- Supplemental\_Fig\_S4.pdf
- Supplemental\_Fig\_S5.pdf
- Supplemental\_Fig\_S6.pdf
- Supplemental\_Fig\_S7.pdf
- Supplemental\_Fig\_S8.pdf
- Supplemental\_Fig\_S9.pdf
- Supplemental\_Fig\_S10.pdf
- Supplemental\_Fig\_S11.pdf
- Supplemental\_Fig\_S12.pdf
- Supplemental\_Fig\_S13.pdf
- Supplemental\_Fig\_S14.pdf
- Supplemental\_Fig\_S15.pdf
- Supplemental\_Fig\_S16.pdf
- Supplemental\_Fig\_S17.pdf
- Supplemental\_Fig\_S18.pdf
- Supplemental\_Fig\_S19.pdf
- Supplemental\_Fig\_S20.pdf
- Supplemental\_Fig\_S21.pdf
- Supplemental\_Fig\_S22.pdf
- Supplemental\_Fig\_S23.pdf
- Supplemental\_Fig\_S24.pdf
- Supplemental\_Fig\_S25.pdf
- Supplemental\_Table\_S6.docx
- Supplemental\_Table\_S7.docx
- Supplemental\_master.zip
- Supplemental\_Table\_S3.docx
- Supplemental\_Table\_S1.docx
- Supplemental\_Table\_S4.docx
- Supplemental\_Table\_S2.docx
- Supplemental\_Table\_S5.docx
