# Supplementary material for: Detection of structural mosaicism from targeted and whole-genome sequencing data
Source: Genome Res. 2017 Oct;27(10):1704–14. doi: 10.1101/gr.212373.116 (PMC5630034; doi:10.1101/gr.212373.116)
Supplement: Supplemental Material [file supp_gr.212373.116_Supplemental_Note_S1.docx]

SUPPLEMENTARY NOTE

Detection of structural mosaicism from targeted and whole-genome sequencing data

Daniel A. King^1^, Alejandro Sifrim^1^, Tomas W. Fitzgerald^1^, Raheleh Rahbari^1^, Emma Hobson^2^, Tessa Homfray^3^, Sahar Mansour^3^, Sarju G. Mehta^4^, Mohammed Shehla^5^, Susan E. Tomkins^6^, Pradeep C. Vasudevan^7^, Matthew E. Hurles^1^, The Deciphering Developmental Disorders Study

^1^ Wellcome Trust Sanger Institute, Hinxton, Cambridge, United Kingdom

^2^ Department of Clinical Genetics, Chapel Allerton Hospital, Leeds, United Kingdom

^3^ Southwest Thames Regional Genetics Centre, St George's Healthcare NHS Trust, London, United Kingdom

^4^ East Anglian Regional Genetics Service, Addenbrookes Hospital, Cambridge, United Kingdom

^5^ Guy's Hospital, London, United Kingdom

^6^ Department of Clinical Genetics, St Michael’s Hospital, Bristol, United Kingdom

^7^ Leicester Royal Infirmary, Leicester, United Kingdom

Daniel A. King: dk6@sanger.ac.uk

Alejandro Sifrim: as33@sanger.ac.uk

Tomas W. Fitzgerald: [tomas@ebi.ac.uk](mailto:tomas@ebi.ac.uk)

Raheleh Rahbari: rr11@sanger.ac.uk

Emma.hobson: [emma.hobson@nhs.net](mailto:emma.hobson@nhs.net)

Tessa Homfray: [thomfray@sgul.ac.uk](mailto:thomfray@sgul.ac.uk)

Sahar Mansour: smansour@sgul.ac.uk

Sarju G Mehta: [sarju.mehta@addenbrookes.nhs.uk](mailto:sarju.mehta@addenbrookes.nhs.uk)

Mohammed Shehla: Shehla.Mohammed@gstt.nhs.uk

Susan E. Tomkins: Susan.Tomkins@UHBristol.nhs.uk

Pradeep C. Vasudevan: [pradeep.vasudevan@uhl-tr.nhs.uk](mailto:pradeep.vasudevan@uhl-tr.nhs.uk)

Matthew E. Hurles: [meh@sanger.ac.uk](mailto:meh@sanger.ac.uk)

# Clinical Assessment

We investigated the clinical impact of the detected mosaic mutations to determine whether each was diagnostic, that is, providing the likely explanation of the child’s phenotype. In an earlier SNP microarray analysis[^1^](#_ENREF_1), we investigated four (Decipher IDs: 261373, 259003, 260462, and 257978) of the nine mutations presented here and the mutations in three of the four children were considered diagnostic of the child’s disease (three multi-megabase mosaic CNVs causing genomic disorders). One child (257978) with a mosaic LOH mutation, had absence of neuronal migration, seizures, somnolence, scoliosis, but no loss of function variants or functional variants in known DD genes in the LOH region (Supplementary Table 5), and the mosaic LOH was considered of uncertain pathogenicity. We investigated the phenotypic profile of the remaining five patients and present the results from that analysis here; we assessed that the mosaic mutation is the likely explanation for disease in each of these children. We summarise the diagnostic results in the following table (Supplementary Table 3) and discuss each patient in detail below.

Female patient 265800 had feeding problems, hypotonia, moderate developmental delay, severe speech delay, joint laxity, macroglossia, meningocele, delayed closure of the anterior fontanelle with short stature (2^nd^ centile). An array CGH was performed on blood lymphocytes but no copy number events were detected. Additionally, testing for mucopolysaccharidosis, SMARCA2, Fragile X, and FISH for 17p11.2 were negative. The exome analysis on saliva detected a gain of 12p. Mosaic tetrasomy 12p is the genetic basis of Pallister Killian syndrome[^2^](#_ENREF_2), a well known cause of developmental delay . Simultaneous skin biopsy confirmed mosaicism for isochromosome 12p. The child’s clinical features are consistent with Pallister Killian and the diagnosis was conferred to the family.

Male patient 273553 has moderate developmental delay, proportionate short stature, mild dysmorphism, significant behavior problems, undescended testes, strabismus, hypermetropia, joint laxity, indistinct speech, palatal insufficiency and communication difficulties. He had surgical correction of a patent ductus arteriosis. Multiple clinical array CGH investigations were performed on blood and all were negative. Exome analysis of saliva detected a mosaic abnormality of 18p, and the abnormality was validated using SNP analysis of saliva (clinical aCGH of the saliva is pending). The gain in chromosome 18 appears to have two extra haplotypes, which may be consistent with a mosaic trisomy condition. Tetrasomy 18p is a recognized genomic disorder, responsible for causing a variety of clinical symptoms. The mosaic form, mosaic tetrasomy 18 presents with milder phenotypes[^3^](#_ENREF_3). In this case, the phenotypes present in the child were considered likely to be due to this mosaic chromosomal abnormality and the diagnosis was conferred to the family.

Male patient 274013 required 35 days of neonatal medical intensive care for feeding difficulties. The child developed with severely restricted growth (1^st^ centile, below -3.5 standard deviations of height, weight, and head circumference) and developmental delay, characterized by severe expressive language disorder and dyspraxia. The child had an abnormal facial shape, abnormal facial musculature, joint stiffness, brachydactyly, short stature, and was mildly dysmorphic. Testing was performed for acroosteolysis and was negative. Clinical array CGH performed in blood was negative. Exome analysis of saliva detected a 13 Mb mosaic deletion affecting the nearly all of 10q26 (10q26.12-10qter). Deletions of 10q26 are responsible for a variety of phenotypes, most commonly pre- and post-natal growth restriction, mental retardation, and abnormal facial facies (broad ‘beak-like’ nose)[^4^](#_ENREF_4). This mosaic abnormality was considered diagnostic and returned to the family.

Female patient 274600 had severe global developmental delay, with absent speech at 5 years of age, severe and progressive microcephaly (below -3.5 standard deviations), muscular hypotonia, hypotelorism, brachycephaly, narrow palate, apneas as a baby, abnormal extensor posturing, beaked nose, bow-shaped upper lip, broad terminal phalanges, and lack of intracranial myelination. Pitt Hopkins was suspected but clinical testing for mutations in the *TCF4* gene, the cause of Pitt Hopkins[^5^](#_ENREF_5) were normal. Additionally, tests for mutations in *UBE3A*, and for abnormalities in 15q methylation were performed and were normal. Exome analysis of saliva detected a 28 Mb mosaic deletion in 18q, overlapping the *TCF4* gene. The child’s phenotypes are suggestive of Pitt Hopkins disorder and the diagnosis was conferred to the family.

Male patient 274396 had mild global developmental delay with severe growth restriction, including substantial microcephaly (below 7 standard deviations), restricted height (below -3.5 standard deviations) and restricted weight (below -5 standard deviations). The child had several abnormalities including progressive hypo- and hyper- pigmentation of the skin especially in the axilla, groin and neck. Skin wrinkling on dorsum of the hands, sparse & fine hair and a wide mouth were also noted. Dyskeratosis congenita was suspected, premature chromosome condensation testing was performed and showed no abnormalities. Exome analysis of saliva detected a large (about 70 Mb) loss of heterozygosity mosaic abnormality on chromosome 11. The LOH mosaicism was unusual in that the clonality of the event varied with genomic location, with increasing clonality towards the telomere. We investigated this region for a suspected mosaic pathogenic variant and found several gene candidates, including one in *CEP57*, a gene associated with mosaic aneuploidy; details described below (Supplementary Figure 18).

# Deciphering Developmental Disorders (DDD) Study Members

<http://www.ddduk.org/>

| Jeremy F McRae |
| --- |
| Stephen Clayton |
| Tomas W Fitzgerald |
| Joanna Kaplanis |
| Elena Prigmore |
| Diana Rajan |
| Alejandro Sifrim |
| Stuart Aitken |
| Nadia Akawi |
| Mohsan Alvi |
| Kirsty Ambridge |
| Daniel M Barrett |
| Tanya Bayzetinova |
| Philip Jones |
| Wendy D Jones |
| Daniel King |
| Netravathi Krishnappa |
| Laura E Mason |
| Tarjinder Singh |
| Adrian R Tivey |
| Munaza Ahmed |
| Uruj Anjum |
| Hayley Archer |
| Ruth Armstrong |
| Jana Awada |
| Meena Balasubramanian |
| Siddharth Banka |
| Diana Baralle |
| Angela Barnicoat |
| Paul Batstone |
| David Baty |
| Chris Bennett |
| Jonathan Berg |
| Birgitta Bernhard |
| A Paul Bevan |
| Maria Bitner-Glindzicz |
| Edward Blair |
| Moira Blyth |
| David Bohanna |
| Louise Bourdon |
| David Bourn |
| Lisa Bradley |
| Angela Brady |
| Simon Brent |
| Carole Brewer |
| Kate Brunstrom |
| David J Bunyan |
| John Burn |
| Natalie Canham |
| Bruce Castle |
| Kate Chandler |
| Elena Chatzimichali |
| Deirdre Cilliers |
| Angus Clarke |
| Susan Clasper |
| Jill Clayton-Smith |
| Virginia Clowes |
| Andrea Coates |
| Trevor Cole |
| Irina Colgiu |
| Amanda Collins |
| Morag N Collinson |
| Fiona Connell |
| Nicola Cooper |
| Helen Cox |
| Lara Cresswell |
| Gareth Cross |
| Yanick Crow |
| Mariella D'Alessandro |
| Tabib Dabir |
| Rosemarie Davidson |
| Sally Davies |
| Dylan de Vries |
| John Dean |
| Charu Deshpande |
| Gemma Devlin |
| Abhijit Dixit |
| Angus Dobbie |
| Alan Donaldson |
| Dian Donnai |
| Deirdre Donnelly |
| Carina Donnelly |
| Angela Douglas |
| Sofia Douzgou |
| Alexis Duncan |
| Jacqueline Eason |
| Sian Ellard |
| Ian Ellis |
| Frances Elmslie |
| Karenza Evans |
| Sarah Everest |
| Tina Fendick |
| Richard Fisher |
| Frances Flinter |
| Nicola Foulds |
| Andrew Fry |
| Alan Fryer |
| Carol Gardiner |
| Lorraine Gaunt |
| Neeti Ghali |
| Richard Gibbons |
| Harinder Gill |
| Judith Goodship |
| David Goudie |
| Emma Gray |
| Andrew Green |
| Philip Greene |
| Lynn Greenhalgh |
| Susan Gribble |
| Rachel Harrison |
| Lucy Harrison |
| Victoria Harrison |
| Rose Hawkins |
| Liu He |
| Stephen Hellens |
| Alex Henderson |
| Sarah Hewitt |
| Lucy Hildyard |
| Emma Hobson |
| Simon Holden |
| Muriel Holder |
| Susan Holder |
| Georgina Hollingsworth |
| Tessa Homfray |
| Mervyn Humphreys |
| Jane Hurst |
| Ben Hutton |
| Stuart Ingram |
| Melita Irving |
| Lily Islam |
| Andrew Jackson |
| Joanna Jarvis |
| Lucy Jenkins |
| Diana Johnson |
| Elizabeth Jones |
| Dragana Josifova |
| Shelagh Joss |
| Beckie Kaemba |
| Sandra Kazembe |
| Rosemary Kelsell |
| Bronwyn Kerr |
| Helen Kingston |
| Usha Kini |
| Esther Kinning |
| Gail Kirby |
| Claire Kirk |
| Emma Kivuva |
| Alison Kraus |
| Dhavendra Kumar |
| V.K Ajith Kumar |
| Katherine Lachlan |
| Wayne Lam |
| Anne Lampe |
| Caroline Langman |
| Melissa Lees |
| Derek Lim |
| Cheryl Longman |
| Gordon Lowther |
| Sally A Lynch |
| Alex Magee |
| Eddy Maher |
| Alison Male |
| Sahar Mansour |
| Karen Marks |
| Katherine Martin |
| Una Maye |
| Emma McCann |
| Vivienne McConnell |
| Meriel McEntagart |
| Ruth McGowan |
| Kirsten McKay |
| Shane McKee |
| Dominic J McMullan |
| Susan McNerlan |
| Catherine McWilliam |
| Sarju Mehta |
| Kay Metcalfe |
| Anna Middleton |
| Zosia Miedzybrodzka |
| Emma Miles |
| Shehla Mohammed |
| Tara Montgomery |
| David Moore |
| Sian Morgan |
| Jenny Morton |
| Hood Mugalaasi |
| Victoria Murday |
| Helen Murphy |
| Swati Naik |
| Andrea Nemeth |
| Louise Nevitt |
| Ruth Newbury-Ecob |
| Andrew Norman |
| Rosie O'Shea |
| Caroline Ogilvie |
| Kai-Ren Ong |
| Soo-Mi Park |
| Michael J Parker |
| Chirag Patel |
| Joan Paterson |
| Stewart Payne |
| Daniel Perrett |
| Julie Phipps |
| Daniela T Pilz |
| Martin Pollard |
| Caroline Pottinger |
| Joanna Poulton |
| Norman Pratt |
| Katrina Prescott |
| Sue Price |
| Abigail Pridham |
| Annie Procter |
| Hellen Purnell |
| Oliver Quarrell |
| Nicola Ragge |
| Raheleh Rahbari |
| Josh Randall |
| Julia Rankin |
| Lucy Raymond |
| Debbie Rice |
| Leema Robert |
| Eileen Roberts |
| Jonathan Roberts |
| Paul Roberts |
| Gillian Roberts |
| Alison Ross |
| Elisabeth Rosser |
| Anand Saggar |
| Shalaka Samant |
| Julian Sampson |
| Richard Sandford |
| Ajoy Sarkar |
| Susann Schweiger |
| Richard Scott |
| Ingrid Scurr |
| Ann Selby |
| Anneke Seller |
| Cheryl Sequeira |
| Nora Shannon |
| Saba Sharif |
| Charles Shaw-Smith |
| Emma Shearing |
| Debbie Shears |
| Eamonn Sheridan |
| Ingrid Simonic |
| Roldan Singzon |
| Zara Skitt |
| Audrey Smith |
| Kath Smith |
| Sarah Smithson |
| Linda Sneddon |
| Miranda Splitt |
| Miranda Squires |
| Fiona Stewart |
| Helen Stewart |
| Volker Straub |
| Mohnish Suri |
| Vivienne Sutton |
| Ganesh Jawahar Swaminathan |
| Elizabeth Sweeney |
| Kate Tatton-Brown |
| Cat Taylor |
| Rohan Taylor |
| Mark Tein |
| I Karen Temple |
| Jenny Thomson |
| Marc Tischkowitz |
| Susan Tomkins |
| Audrey Torokwa |
| Becky Treacy |
| Claire Turner |
| Peter Turnpenny |
| Carolyn Tysoe |
| Anthony Vandersteen |
| Vinod Varghese |
| Pradeep Vasudevan |
| Parthiban Vijayarangakannan |
| Julie Vogt |
| Emma Wakeling |
| Sarah Wallwark |
| Jonathon Waters |
| Astrid Weber |
| Diana Wellesley |
| Margo Whiteford |
| Sara Widaa |
| Sarah Wilcox |
| Emily Wilkinson |
| Denise Williams |
| Nicola Williams |
| Louise Wilson |
| Geoff Woods |
| Christopher Wragg |
| Michael Wright |
| Laura Yates |
| Michael Yau |
| Chris Nellåker |
| Michael J Parker |
| Helen V Firth |
| Caroline F Wright |
| David R FitzPatrick |
| Jeffrey C Barrett |
| Matthew E Hurles |

# References

1. King, D.A. *et al.* Mosaic structural variation in children with developmental disorders. *Hum Mol Genet* (2015).

2. Reynolds, J.F. *et al.* Isochromosome 12p mosaicism (Pallister mosaic aneuploidy or Pallister-Killian syndrome): report of 11 cases. *Am J Med Genet* 27, 257-74 (1987).

3. Plaiasu, V., Ochiana, D., Motei, G. & Georgescu, A. A rare chromosomal disorder - isochromosome 18p syndrome. *Maedica (Buchar)* 6, 132-6 (2011).

4. Wulfsberg, E.A., Weaver, R.P., Cunniff, C.M., Jones, M.C. & Jones, K.L. Chromosome 10qter deletion syndrome: a review and report of three new cases. *Am J Med Genet* 32, 364-7 (1989).

5. Zweier, C. *et al.* Haploinsufficiency of TCF4 causes syndromal mental retardation with intermittent hyperventilation (Pitt-Hopkins syndrome). *Am J Hum Genet* 80, 994-1001 (2007).

6. Choate, K.A. *et al.* Mitotic recombination in patients with ichthyosis causes reversion of dominant mutations in KRT10. *Science* 330, 94-7 (2010).

7. Abyzov, A., Urban, A.E., Snyder, M. & Gerstein, M. CNVnator: an approach to discover, genotype, and characterize typical and atypical CNVs from family and population genome sequencing. *Genome Res* 21, 974-84 (2011).
