# Supplementary material for: Detection of structural mosaicism from targeted and whole-genome sequencing data
Source: Genome Res. 2017 Oct;27(10):1704–14. doi: 10.1101/gr.212373.116 (PMC5630034; doi:10.1101/gr.212373.116)
Supplement: Supplemental Material [file supp_gr.212373.116_Supplemental_Fig_S1.pdf]

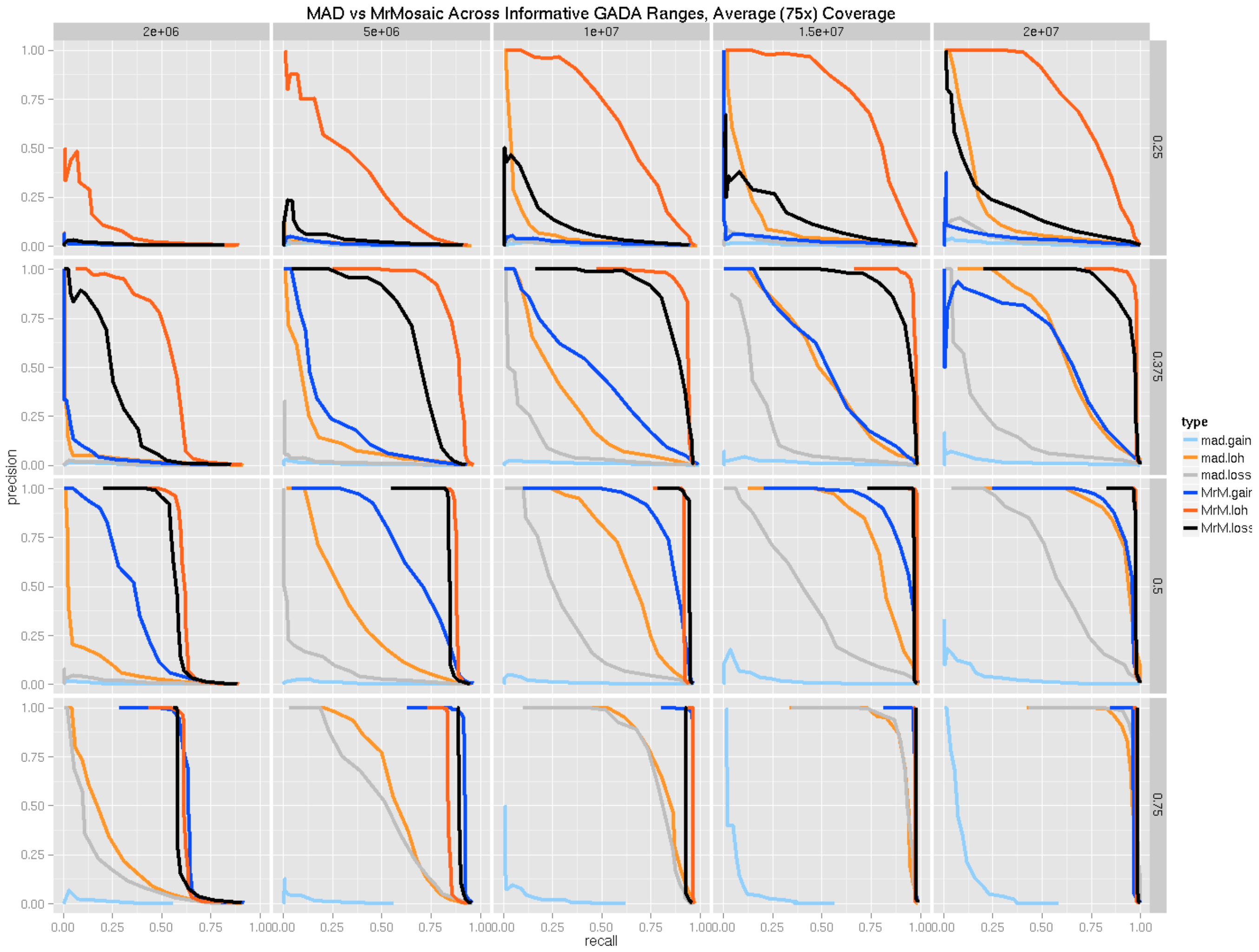

Supplementary Figure 1: WE performance of MAD and MrMosaic algorithms: In this grid of precision-recall graphs, the performance of MAD and MrMosaic is compared at 75x average coverage for a range of sizes, clonalities, and for the three types of mosaic abnormalities. The column header and row header describe the size and derived-clonality of the introduced mosaic event (a deletion of 1.0 clonality indicates hemizygosity). Performance of both algorithms improves with increasing simulated event size (due to more assayed informative points) and at higher clonalities (due to a stronger deflection of non-reference proportion (B_{dev}) and coverage (C_{dev})). MrMosaic performs favorably compared to MAD in all measured categories. This effect is especially apparent for mosaic gains, which is the type of mosaicism that generates he smallest deviations in B_{dev}; unlike MrMosaic, which analyses B_{dev} and C_{dev}, MAD analyses B_{dev} alone.
