# Supplementary material for: Detection of structural mosaicism from targeted and whole-genome sequencing data
Source: Genome Res. 2017 Oct;27(10):1704–14. doi: 10.1101/gr.212373.116 (PMC5630034; doi:10.1101/gr.212373.116)
Supplement: Supplemental Material [file supp_gr.212373.116_Supplemental_Fig_S2.pdf]

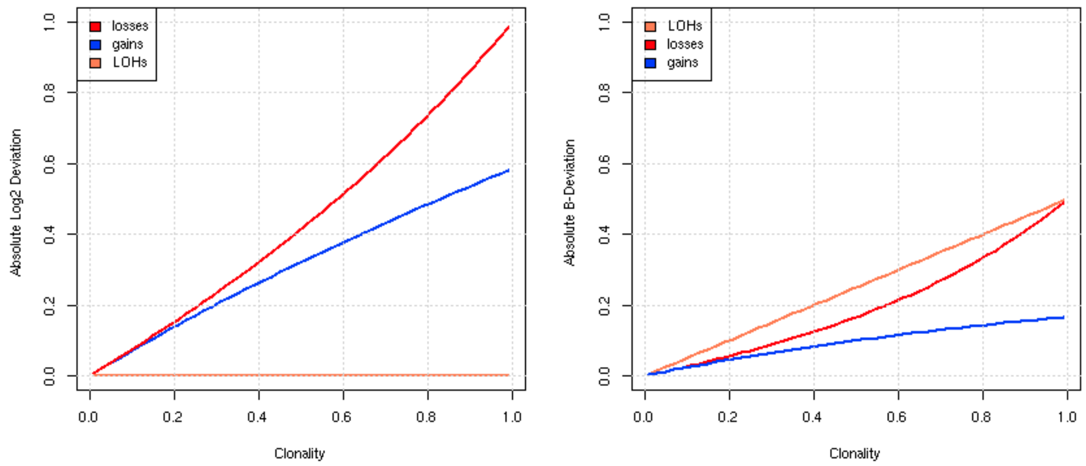

Supplementary Figure 2: Relationship between Clonality and Metrics: The relationship between clonality and measured metrics (C_{dev} and B_{dev}) indicates that while LOH events result in no deviation of C_{dev}, gains have the smallest deflection of B_{dev}, compared to other events of a given clonality.
