# Supplementary material for: Detection of structural mosaicism from targeted and whole-genome sequencing data
Source: Genome Res. 2017 Oct;27(10):1704–14. doi: 10.1101/gr.212373.116 (PMC5630034; doi:10.1101/gr.212373.116)
Supplement: Supplemental Material [file supp_gr.212373.116_Supplemental_Fig_S3.pdf]

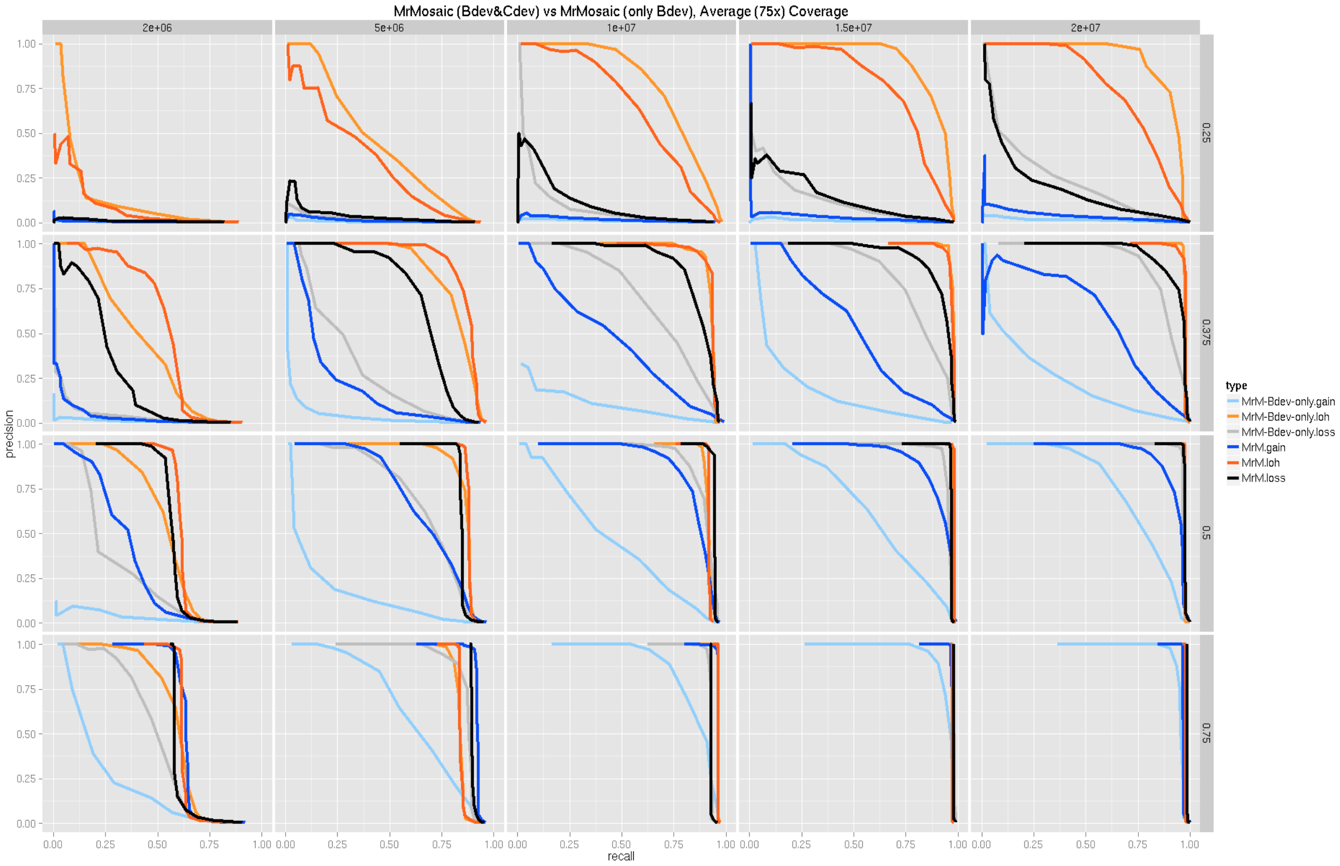

Supplementary Figure 3: WE MrMosaic, Cdev & Bdev vs Bdev-alone: MrMosaic combines the statistical deviation from differences in coverage (C_{dev}) and non-reference proportion (B_{dev}) while the MAD approach uses B_{dev} alone. We ran MrMosaic in standard joint-mode and also using B_{dev} alone. The results demonstrate improved detection when considering joint calling, especially for copy number events above 0.25 clonality. LOH-type mosaicism does not affect copy number (C_{dev}), so considering C_{dev} adds no additional information and has the potential to add noise to the calculation, which may explain the slightly lower performance of LOH calling in the low-clonality (0.25), large (20 Mb) category. This effect is more outspoken for larger event sizes as the recall rate increases. As clonality increases this difference between the combined and the Bdev-only method decreases as long as the signal strength of the Bdev signal is vastly greater than the noise added by combining with the Cdev. For smaller event sizes the combined method uses the coverage information (including neighbouring homozygous sites) to smooth out peaks/drops in coverage and therefore reduces variance which might produce false Bdev signals, resulting in an increase in precision.
