# Supplementary material for: Detection of structural mosaicism from targeted and whole-genome sequencing data
Source: Genome Res. 2017 Oct;27(10):1704–14. doi: 10.1101/gr.212373.116 (PMC5630034; doi:10.1101/gr.212373.116)
Supplement: Supplemental Material [file supp_gr.212373.116_Supplemental_Fig_S4.pdf]

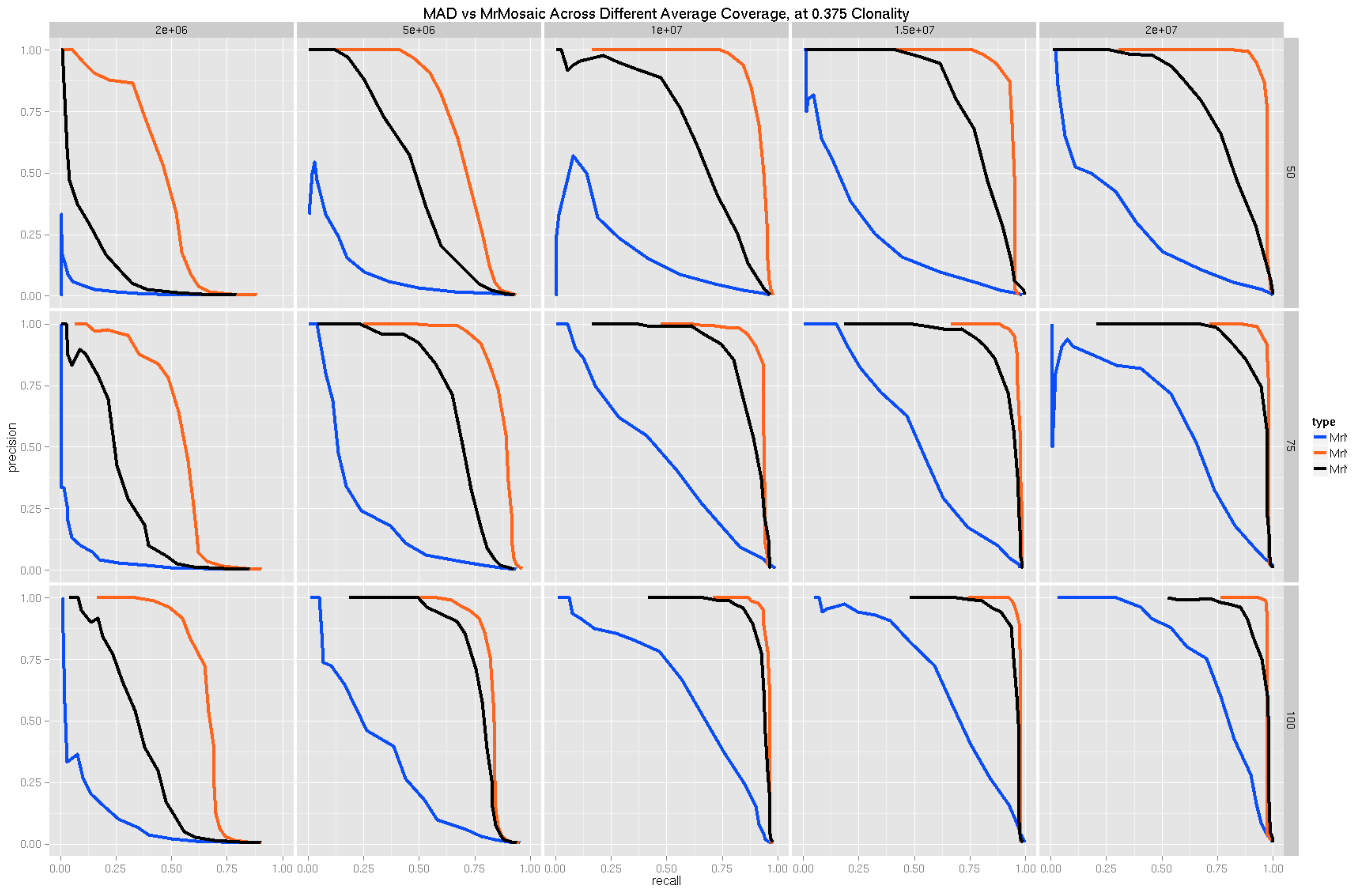

Supplementary Figure 4: WE performance of MrMosaic across 50-100x: We generated simulated exomes of 50x, 75x, and 100x depths and measured MrMosaic detection performance across coverage. Detection was measured at events of 0.5 clonality. Simulated event size and coverage (in 'x') are denoted in column and row headers, respectively. Increasing coverage is positively correlated with higher performance. This is likely due to a greater number of events passing minimum depth threshold (more signals) and a more precise estimate of non-reference discrepancy (better signal:noise ratio).
