# Supplementary material for: Detection of structural mosaicism from targeted and whole-genome sequencing data
Source: Genome Res. 2017 Oct;27(10):1704–14. doi: 10.1101/gr.212373.116 (PMC5630034; doi:10.1101/gr.212373.116)
Supplement: Supplemental Material [file supp_gr.212373.116_Supplemental_Fig_S5.pdf]

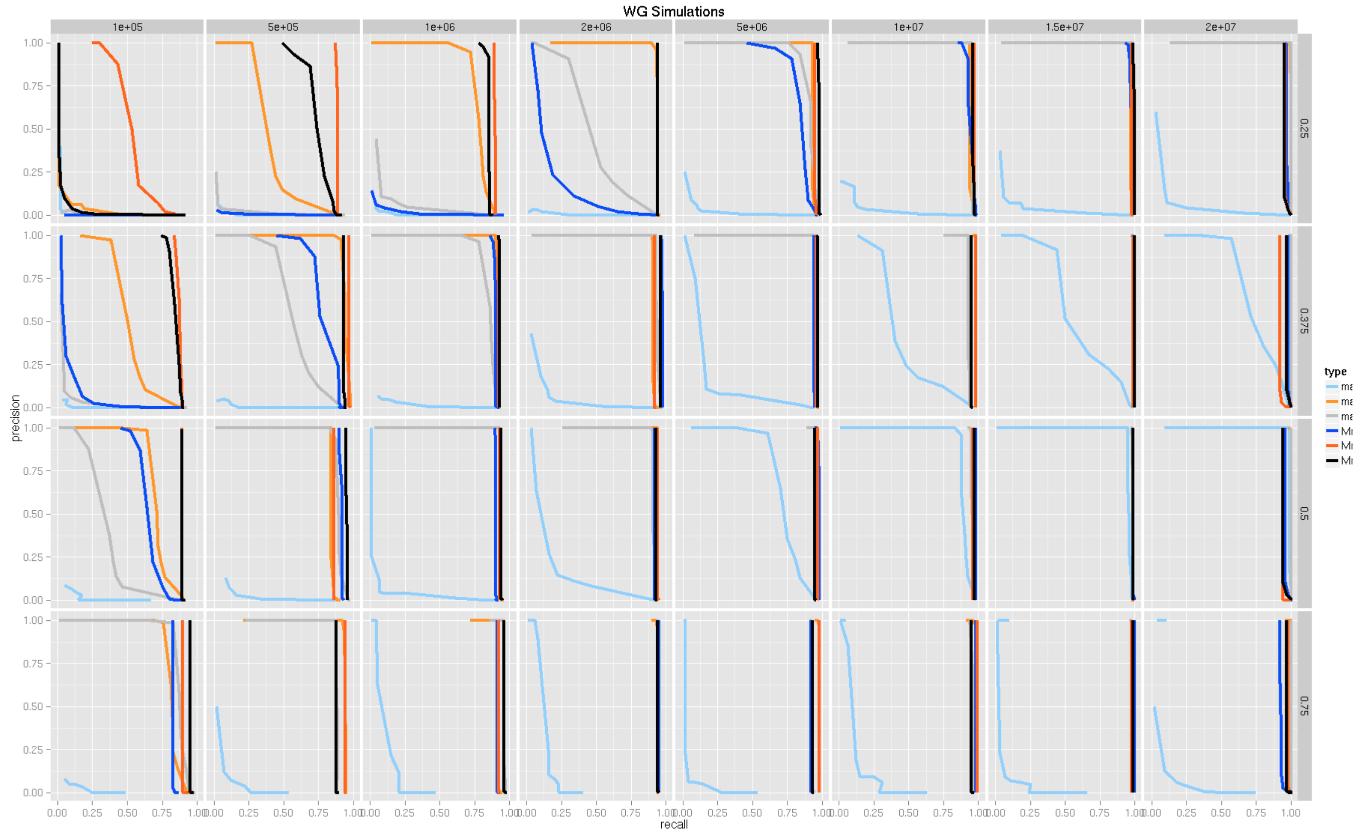

Supplementary Figure 5: WG performance of MrMosaic and MAD: The performance of MAD and MrMosaic is compared at 30x WG average coverage for a range of sizes, clonalities, and for the three types of mosaic abnormalities simulations. The performance of MrMosaic detection is extremely high (high recall, high precision) at the same size ranges (2 Mb to 20 Mb) tested in exome simultions. In addition, detection performance is high at small-sized (100,000 bp) medium-clonality (0.5) events.
