# Supplementary material for: Detection of structural mosaicism from targeted and whole-genome sequencing data
Source: Genome Res. 2017 Oct;27(10):1704–14. doi: 10.1101/gr.212373.116 (PMC5630034; doi:10.1101/gr.212373.116)
Supplement: Supplemental Material [file supp_gr.212373.116_Supplemental_Fig_S6.pdf]

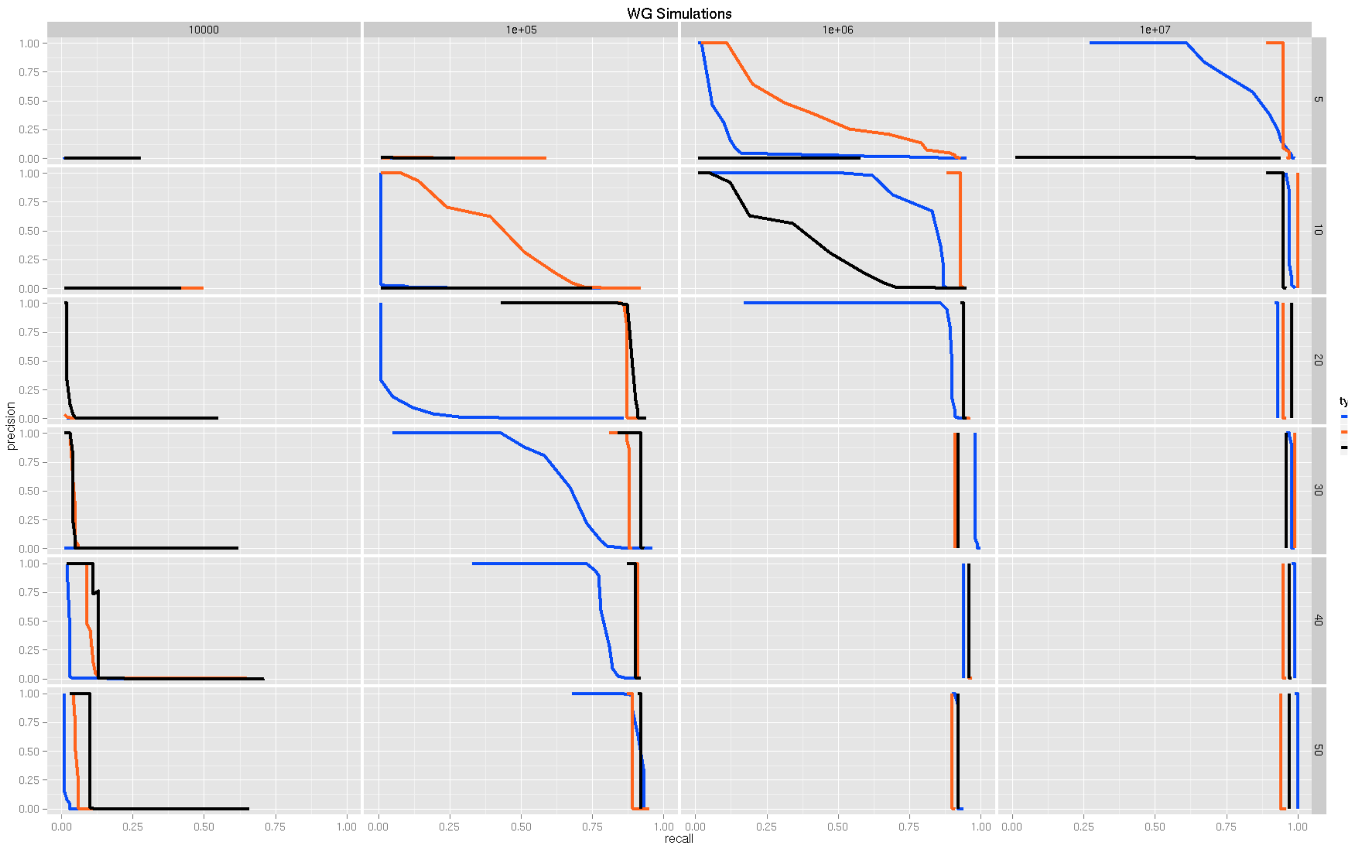

Supplementary Figure 6: WG MrMosaic performance across 5-50x: We generated simulated genomes of 5x-50x depths and measured MrMosaic detection performance across coverage. Performance was measured of simulated events of 0.5 clonality. Simulated event size and coverage (in X) are denoted in column and row headers, respectively. Increasing coverage is positively correlated with higher performance. Events at 1Mb were detected easily at standard X-Ten coverage (30x) (http://www.illumina.com/systems/hiseq-x-sequencing-system/system.html).
