# Supplementary figures and images for: Detection of structural mosaicism from targeted and whole-genome sequencing data
Source: Genome Res. 2017 Oct;27(10):1704–14. doi: 10.1101/gr.212373.116 (PMC5630034; doi:10.1101/gr.212373.116)

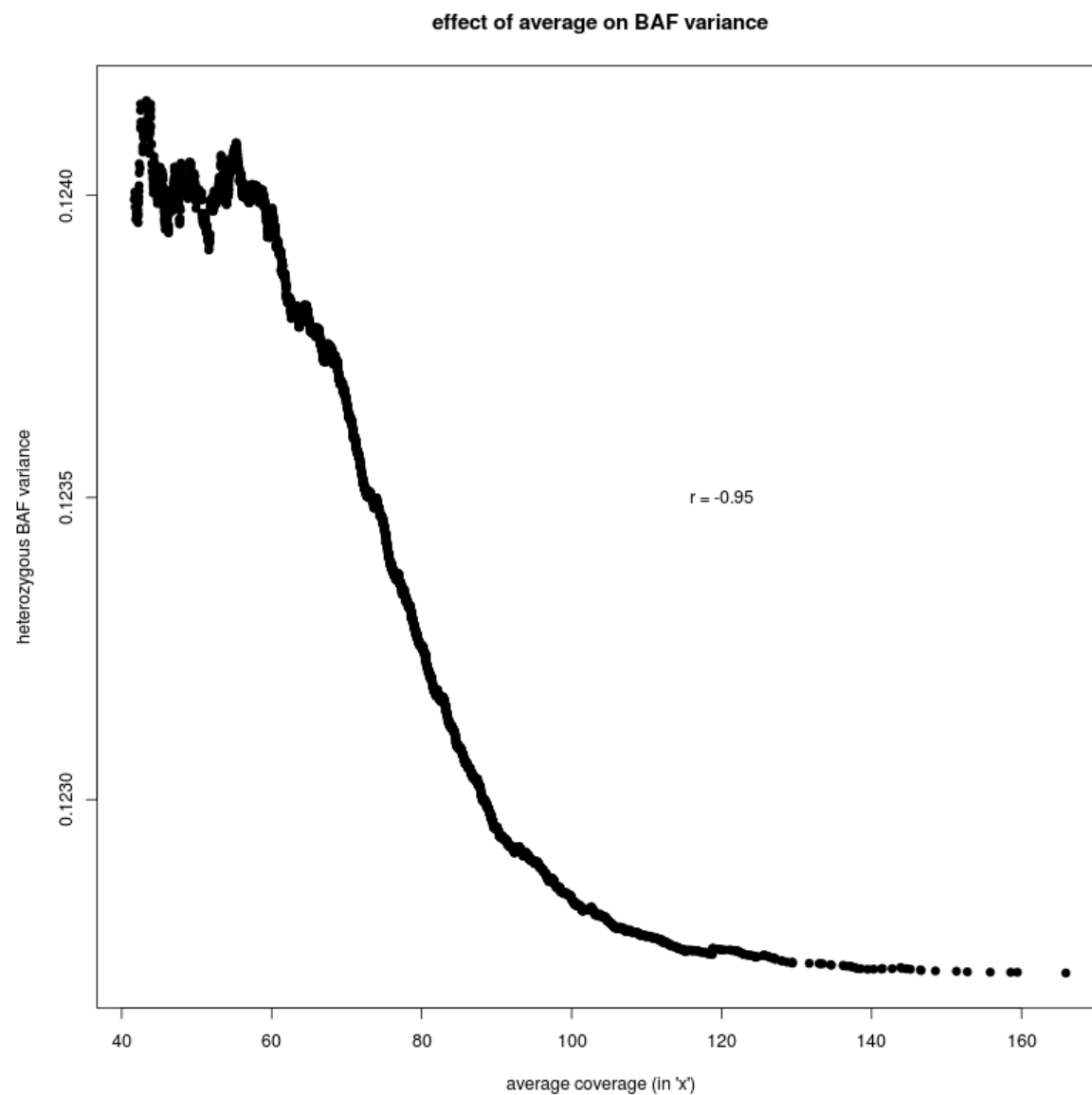

**Supplementary Figure 7: The effect of average coverage on BAF variance.**

Supplement: Supplemental Material [file supp_gr.212373.116_Supplemental_Fig_S7.pdf]
