# Supplementary material for: Detection of structural mosaicism from targeted and whole-genome sequencing data
Source: Genome Res. 2017 Oct;27(10):1704–14. doi: 10.1101/gr.212373.116 (PMC5630034; doi:10.1101/gr.212373.116)
Supplement: Supplemental Material [file supp_gr.212373.116_Supplemental_Fig_S17.pdf]

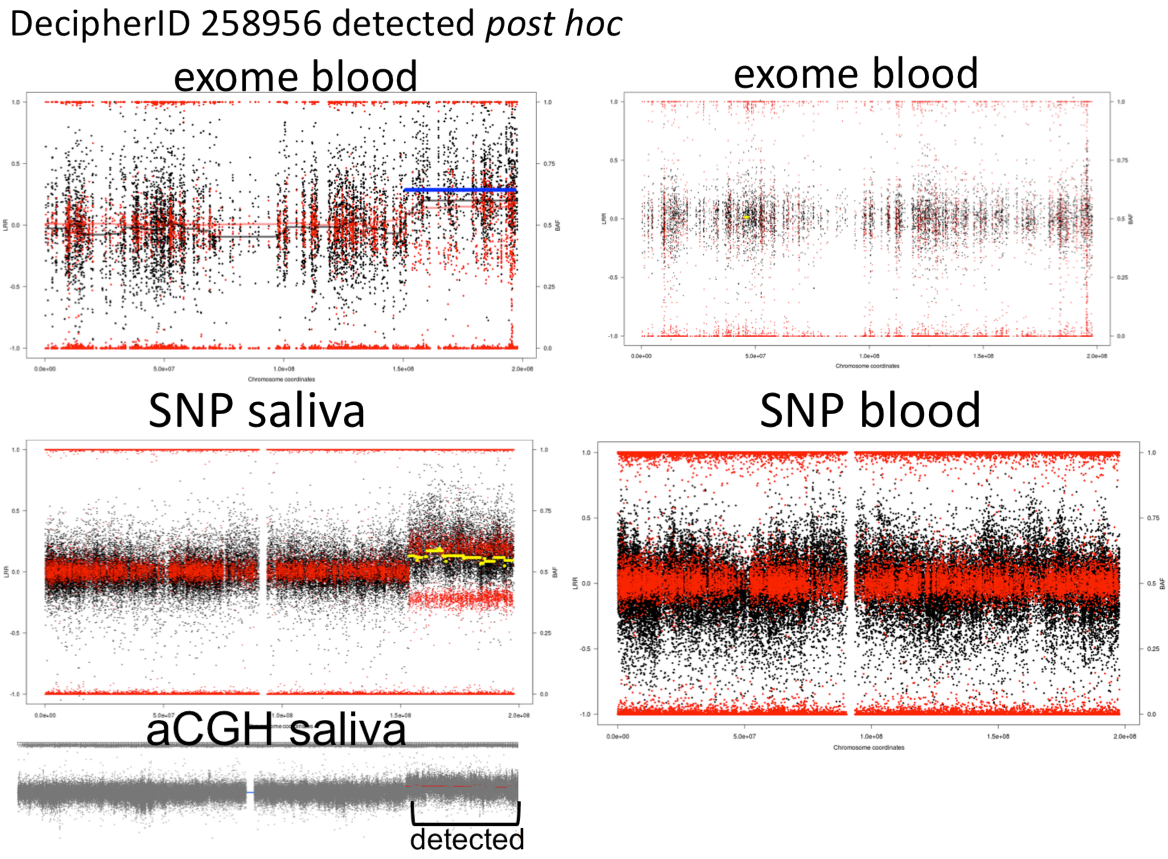

Supplementary Figure 17: Post-hoc detection of sample 258956: Initially undetected in exome (done on blood), SNP-chip results showed that the event is absent in blood. Post-hoc detection, by exome, of saliva successfully detected the event. The Mscore of this detection was 19.
