# Supplementary material for: Detection of structural mosaicism from targeted and whole-genome sequencing data
Source: Genome Res. 2017 Oct;27(10):1704–14. doi: 10.1101/gr.212373.116 (PMC5630034; doi:10.1101/gr.212373.116)
Supplement: Supplemental Material [file supp_gr.212373.116_Supplemental_Fig_S18.pdf]

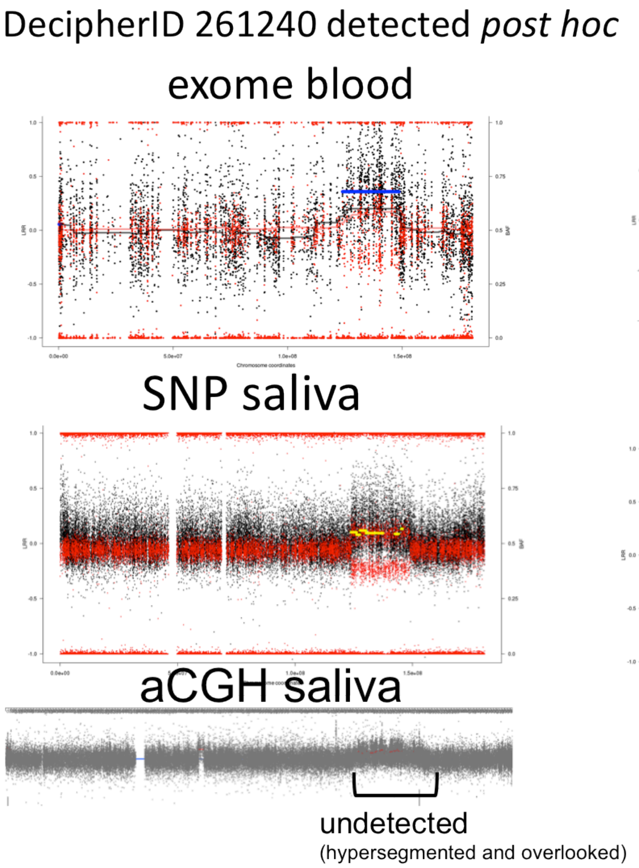

Supplementary Figure 18: Post-hoc detection of sample 261240: Initially undetected in exome (done on blood), SNP-chip results showed that the event is absent in blood. Post-hoc detection, by exome, of saliva successfully detected the event. Note that array CGH of saliva identified small segments of elevation but none was sufficiently large to pass size filtering. The Mscore of this detection was 12.
