# Supplementary material for: Detection of structural mosaicism from targeted and whole-genome sequencing data
Source: Genome Res. 2017 Oct;27(10):1704–14. doi: 10.1101/gr.212373.116 (PMC5630034; doi:10.1101/gr.212373.116)
Supplement: Supplemental Material [file supp_gr.212373.116_Supplemental_Fig_S19.pdf]

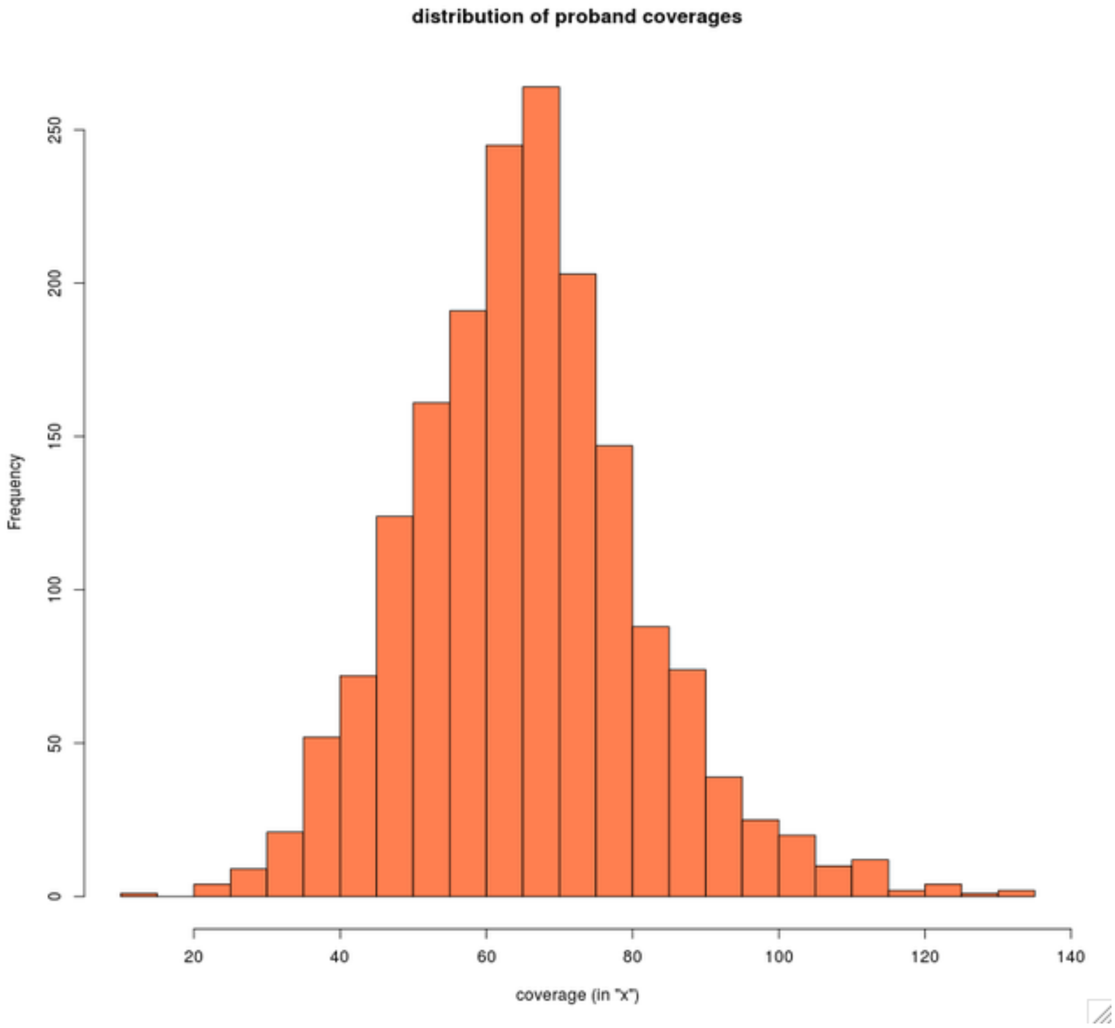

Supplementary Figure 19: Distribution of proband coverages: We measured coverage in exomes using samtools flagstat to generate the number QC-passed and mapped reads without read-duplicates, multipled this by the sequence read length (75) and divided by the number of bases targeted in the exome, 90 Mb. The mean exome coverage was 69x.
