# Supplementary material for: Detection of structural mosaicism from targeted and whole-genome sequencing data
Source: Genome Res. 2017 Oct;27(10):1704–14. doi: 10.1101/gr.212373.116 (PMC5630034; doi:10.1101/gr.212373.116)
Supplement: Supplemental Material [file supp_gr.212373.116_Supplemental_Fig_S20.pdf]

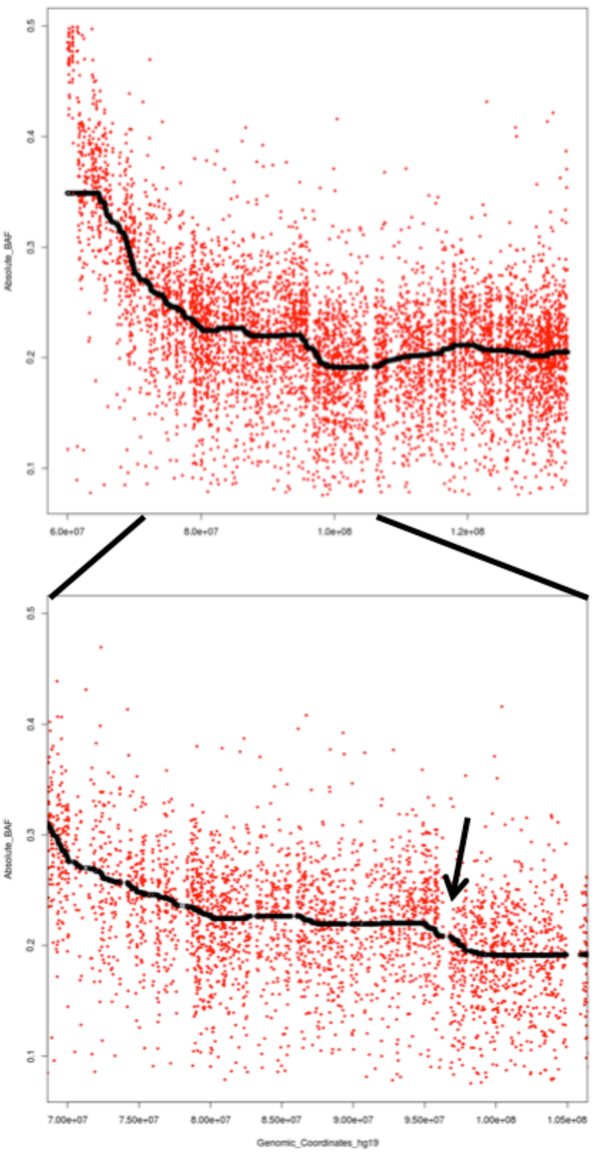

Supplementary Figure 20: Mosaic reversion event: We examined SNP microarray data to help localise the cause of the suspected reversion. These plots displays heterozygous BAFs (BAFs above 0.5 are reflected below the 0.5 line) from SNP microarray data on the 3' end of chromosome 11, with a median trend line included. The bottom plot is a zoomed-in version of the top plot. Just 5' to the 100 Mb position there is a sudden increase in mosaic clonality (arrow), followed by a plateau of clonality toward the 3' end. In 2010 Choate *et al.*[6] biopsied normal-appearing skin in a patient with ichthyosis, identified that each biopsied normal tissue represented a different LOH-mediated reversion clone generated from somatic recombination, overlaid the LOH segments present among the clones, and used the overlapping breakpoints to map the location of a *de novo* point mutation in the *KRT10* gene to the point immediately distal to the shortest LOH segment. In our sample, the gradient of mosaicism is thought to reflect many overlapping LOH regions, and the arrow specifies the location of the end of the shortest LOH region in our sample. Therefore, we scrutinised the genomic interval in the most proximal (5') portion of this LOH segment (just distal to the arrow). This location harbours the *CEP57* gene, between GRCh37 coordinates 95,523,625 - 95,565,857, a gene implicated in a mosaic condition, mosaic aneuploidy syndrome, and thus is an excellent candidate for the presence of a mutation underlying this child's mosaicism. We investigated the rare (below 1%) variants present in the region from 90 Mb – 105 Mb and present the results in the following table (Supplementary Table). Interrogation of high-depth exome data in this gene and the surrounding genome identified no loss-of-function or functional variants in this area. A rare (0.71%) intronic mutation of uncertain significance was found in the *CEP57* gene.
