# Supplementary material for: Detection of structural mosaicism from targeted and whole-genome sequencing data
Source: Genome Res. 2017 Oct;27(10):1704–14. doi: 10.1101/gr.212373.116 (PMC5630034; doi:10.1101/gr.212373.116)
Supplement: Supplemental Material [file supp_gr.212373.116_Supplemental_Fig_S21.pdf]

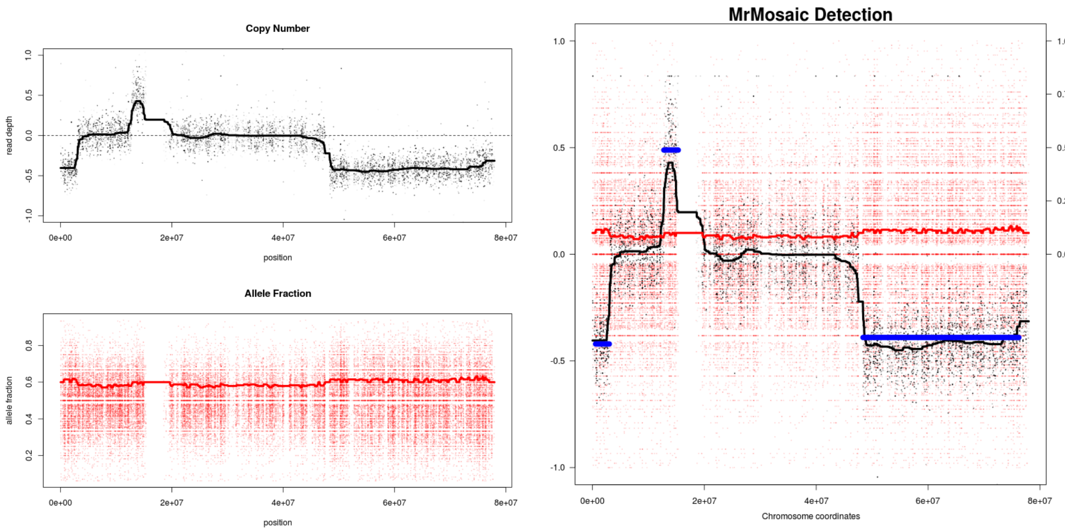

Supplementary Figure 21: WGS analysis of Decipher sample 260462: Measurement of copy number (left, top) was generated using CNVnator[7], using bins of 10k reads and normalizing by GC content. CNVnator stores metadata in a *root* object and histogram data were extracted using this code* and depth was processed using the *scale* function in R of the histogram data. The allele fraction plot (left, bottom) shows slight more variance in BAFs at the termini of the chromosomes. MrMosaic detection (Tgada of 20, minSegLen of 30) identified the three mosaic abnormalities (blue lines). The BAF signal is 'noiser' here than in the exome analysis because measurement of BAF is sensitive to sampling variance, which is related to read coverage, and coverage is much lower in the WGS (25x) compared to the WES data (75x).

***https://github.com/findingdan/small_scripts/hist_to_text.root_commands.10k.txt**
