# Supplementary material for: Detection of structural mosaicism from targeted and whole-genome sequencing data
Source: Genome Res. 2017 Oct;27(10):1704–14. doi: 10.1101/gr.212373.116 (PMC5630034; doi:10.1101/gr.212373.116)
Supplement: Supplemental Material [file supp_gr.212373.116_Supplemental_Fig_S22.pdf]

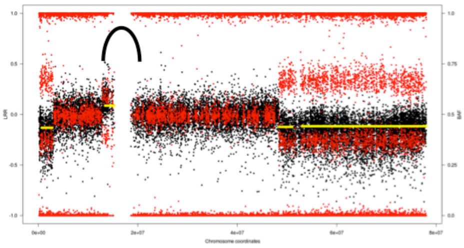

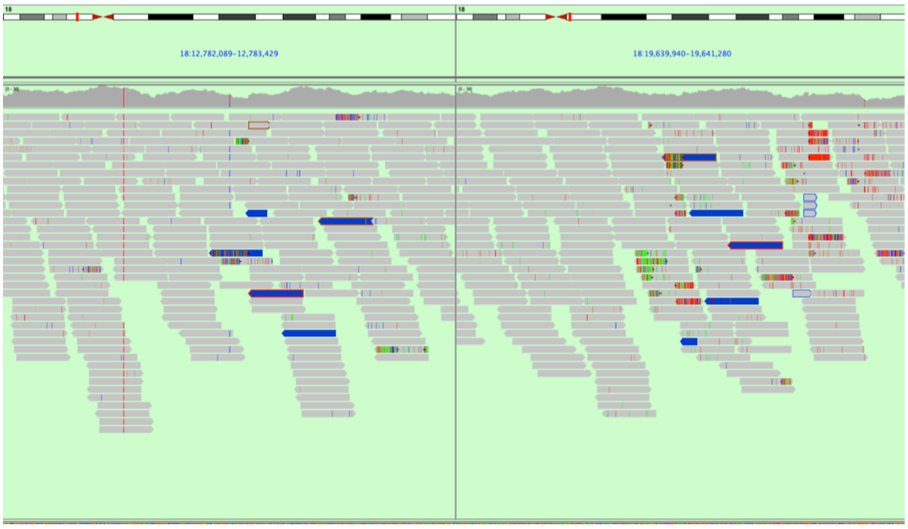

Chr1	Pos1	Orientation1	Chr2	Pos2	Orientation2	Type	Size	Score	num_Reads	AF
18	12783163	0+5-	18	19640612	1+5-	INV	6857381	99	4	0.0519481

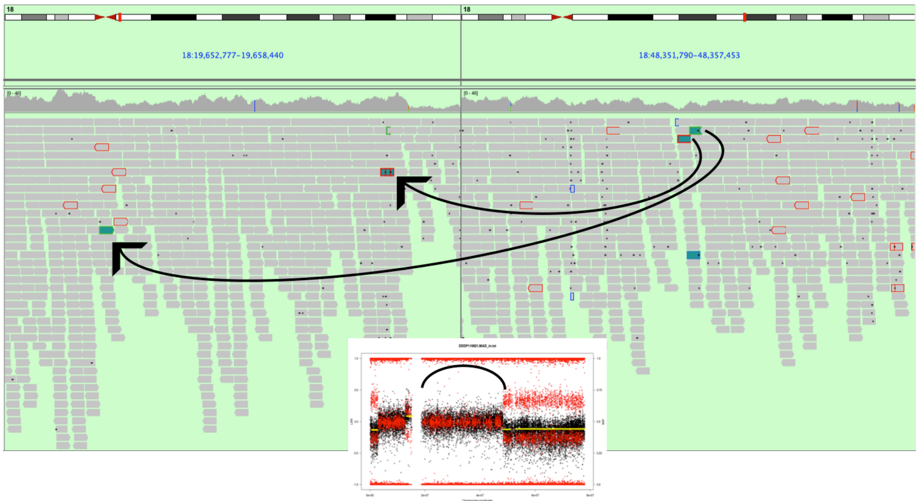

Supplementary Figure 22: Investigation of read-pair data for the WGS sample: Analysis of read pair information in the WGS data of this sample identified a cluster of four aberrantly read pairs mapping to different sides of the centromere. Using the structural variation detection tool Breakdancer, these read pairs were 'called' as an inversion rearrangement. Given the read depth and mosaicism, this may reflect an inverted duplication. Weaker read pair evidence also supported the existence of a rearrangement between 19.655 Mb and 48.355 Mb. No aberrant mappings were observed at the boundary of the 5' p-arm event (at ~3Mb).
