# Supplementary material for: Detection of structural mosaicism from targeted and whole-genome sequencing data
Source: Genome Res. 2017 Oct;27(10):1704–14. doi: 10.1101/gr.212373.116 (PMC5630034; doi:10.1101/gr.212373.116)
Supplement: Supplemental Material [file supp_gr.212373.116_Supplemental_Fig_S23.pdf]

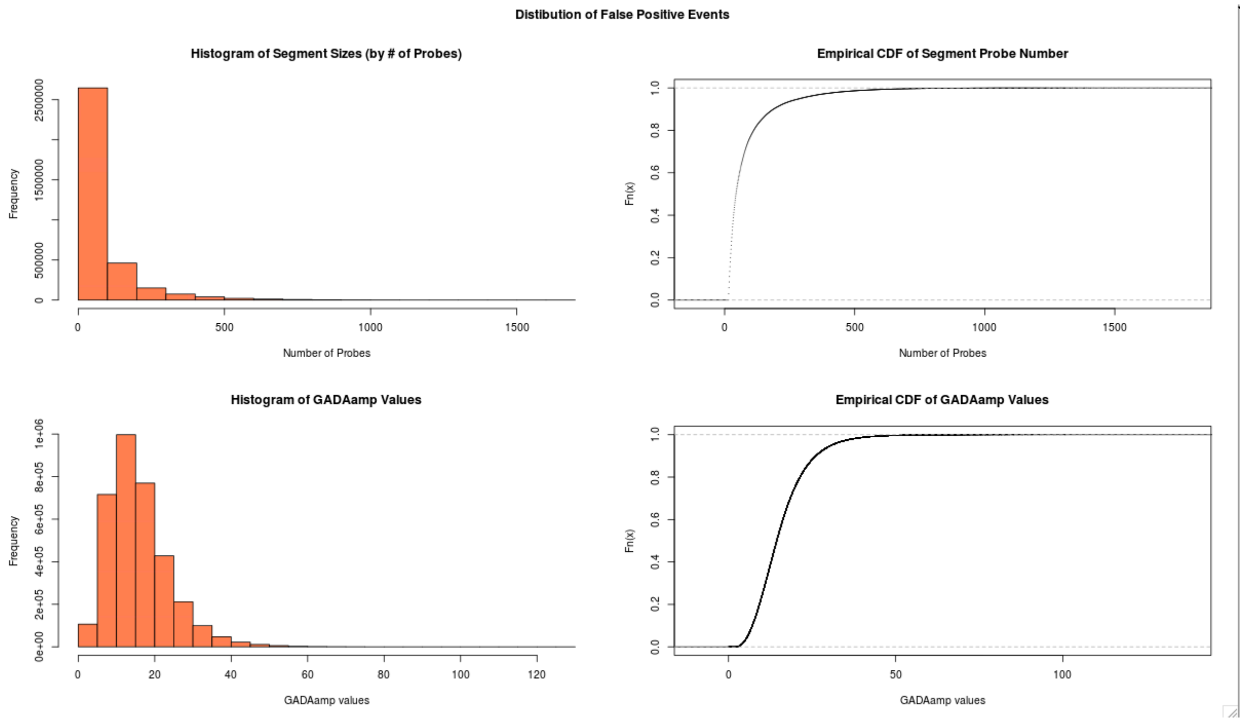

Supplementary Figure 23: Distribution of size and signal-strength of false positives: The histograms of probe-number and GADAamp values both show long tails, with the majority of putative events being smaller and weak. We plotted the cumulative distribution functions from the data and showed that events with greater than about 100 probes or about 25 GADAamp were very rare in the false positive events.
