# Supplementary material for: Detection of structural mosaicism from targeted and whole-genome sequencing data
Source: Genome Res. 2017 Oct;27(10):1704–14. doi: 10.1101/gr.212373.116 (PMC5630034; doi:10.1101/gr.212373.116)
Supplement: Supplemental Material [file supp_gr.212373.116_Supplemental_Fig_S24.pdf]

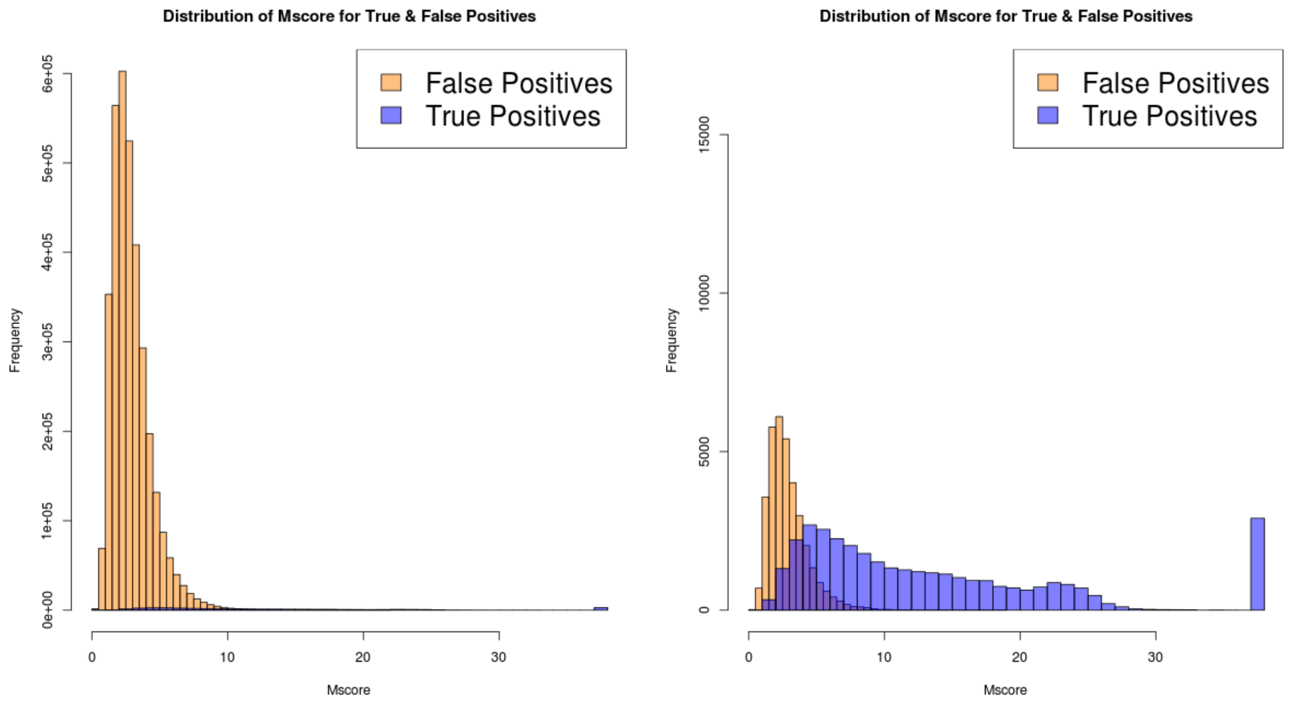

Supplementary Figure 24: Comparing Mscores of true positives and false positives: The Mscore distributions for all simulated false positive events (left graph) and for a random subselection of false positive events equal to the number of true positive events (right graph) demonstrates that the true positive events in general have higher Mscores. The accumulation of true positive events at ~40 is an artefact of assigning a maximum cut-off to an R "-Inf" value.
