# Supplementary material for: Detection of structural mosaicism from targeted and whole-genome sequencing data
Source: Genome Res. 2017 Oct;27(10):1704–14. doi: 10.1101/gr.212373.116 (PMC5630034; doi:10.1101/gr.212373.116)
Supplement: Supplemental Material [file supp_gr.212373.116_Supplemental_Fig_S25.pdf]

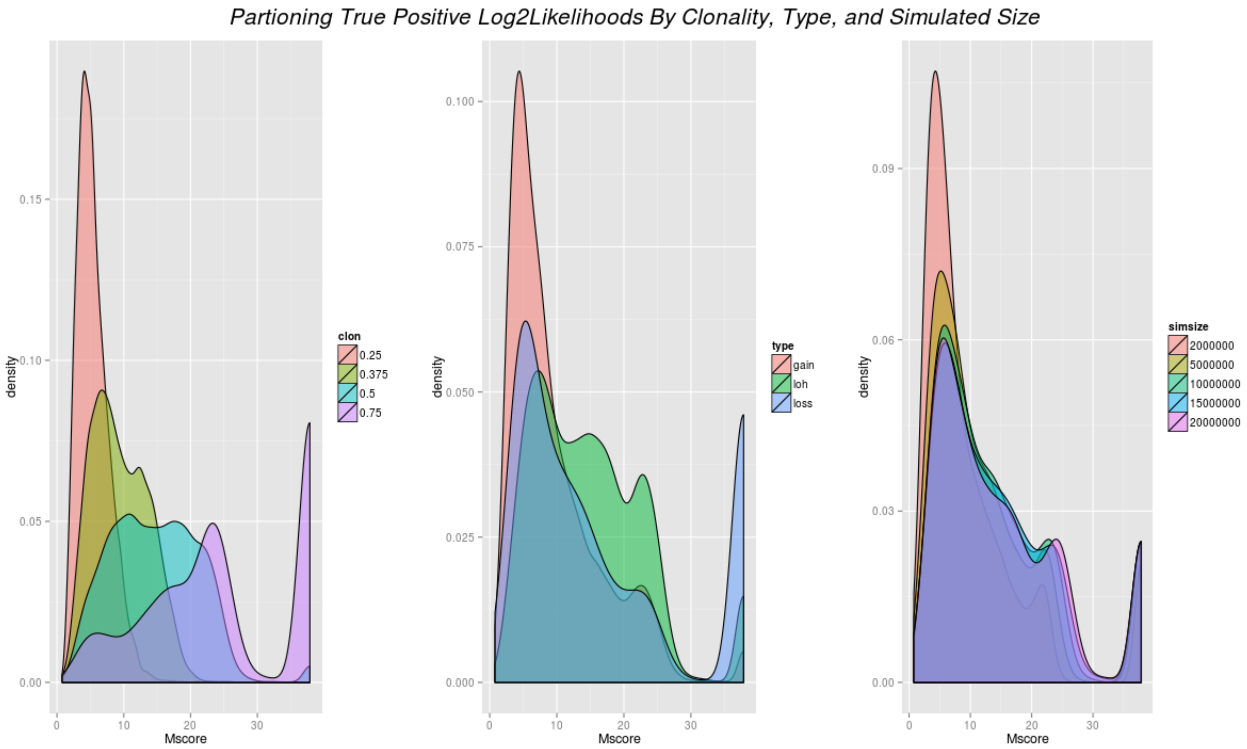

Supplementary Figure 25: Stratifying Mscore by simulation clonality, type, and size: We stratified the true positive events by Mscore to better define the relationship between Mscore thresholds and simulated mosaic events. The mosaic events with the lowest Mscore were those at the lowest clonality (left side of left graph).
