# Supplementary material for: Detection of structural mosaicism from targeted and whole-genome sequencing data
Source: Genome Res. 2017 Oct;27(10):1704–14. doi: 10.1101/gr.212373.116 (PMC5630034; doi:10.1101/gr.212373.116)
Supplement: Supplemental Material [file supp_gr.212373.116_Supplemental_Table_S6.docx]

Supplementary Table 6: Functions to Prepare Simulations

| Simulation metrics | Normal | Loss | Gain | LOH |
| --- | --- | --- | --- | --- |
| LRR | 0 | $log2(\frac{2-m}{2})$ | $log2(\frac{2+m}{2})$ | 0 |
| Simulated Read Depth (SDP) | $\lambda_{i}=\tilde{{DP}_{i}}\cdot S$  ${SDP}_{i}\sim Poiss(\lambda_{i})$ | $\lambda_{i}=\tilde{{DP}_{i}}\left( \frac{2-m}{2} \right)S$  ${SDP}_{i}\sim Poiss(\lambda_{i})$ | $\lambda_{i}=\tilde{{DP}_{i}}\left( \frac{2+m}{2} \right)S$  ${SDP}_{i}\sim Poiss(\lambda_{i})$ | $\lambda_{i}=\tilde{{DP}_{i}}\cdot S$  ${SDP}_{i}\sim Poiss(\lambda_{i})$ |
| B-allele frequency  (B_dev_) | $p=0.5$  $B_{dev,i}\sim Binom({SDP}_{i},p_{i})$ | $p=0.5\pm\frac{m}{2(2-m)}$  $B_{dev,i}\sim Binom({SDP}_{i},p_{i})$ | $p=0.5\pm\frac{m}{2(2+m)}$  $B_{dev,i}\sim Binom({SDP}_{i},p_{i})$ | $p=0.5\pm\frac{m}{2}$  $B_{dev,i}\sim Binom({SDP}_{i},p_{i})$ |

**Legend:**

$m$*:* Clonality as in proportion of cells with abnormality

$\tilde{{DP}_{i}}$*:* Median read depth (after quality filtering) at position *i*

*S*: Scaling factor so that $Target Average Read Depth = 75.2 \times S$

SDP_i_ : Simulated Read Depth at position *i*

p: Proportion of reads with alternative allele at position *i*
