# Supplementary material for: Detection of structural mosaicism from targeted and whole-genome sequencing data
Source: Genome Res. 2017 Oct;27(10):1704–14. doi: 10.1101/gr.212373.116 (PMC5630034; doi:10.1101/gr.212373.116)
Supplement: Supplemental Material [file supp_gr.212373.116_Supplemental_Table_S7.docx]

Supplementary Table 7: Functions to Relate Clonality to Bdev: Relationship between clonality and absolute deviation in coverage (C_dev_) non-reference fraction (B_dev_). Copy number events result in deviation in both Cdev and Bdev.

| Type | Function of b_dev_ |
| --- | --- |
| Loss | $m=\frac{2(Bdev)}{0.5+Bdev}$ |
| Gain | $m=\frac{2(Bdev)}{0.5-Bdev}$ |
| LOH | $m=2 (Bdev)$ |

**Legend:**

$m$*:* Clonality as in proportion of cells with abnormality

*Bdev*: value of B_dev_
