# Supplementary material for: Detection of structural mosaicism from targeted and whole-genome sequencing data
Source: Genome Res. 2017 Oct;27(10):1704–14. doi: 10.1101/gr.212373.116 (PMC5630034; doi:10.1101/gr.212373.116)
Supplement: Supplemental Material [file supp_gr.212373.116_Supplemental_Table_S3.docx]

Supplementary Table 3 Diagnoses

| **DecipherID** | **Diagnosis** |
| --- | --- |
| 265800 | Pallister Killian syndrome |
| 273553 | 18p mosaic tetrasomy |
| 274013 | distal 10q deletion syndrome |
| 274600 | Pitt Hopkins syndrome |
| 274396 | mosaic reversion of unknown de novo mutation |
