# Supplementary material for: Detection of structural mosaicism from targeted and whole-genome sequencing data
Source: Genome Res. 2017 Oct;27(10):1704–14. doi: 10.1101/gr.212373.116 (PMC5630034; doi:10.1101/gr.212373.116)
Supplement: Supplemental Material [file supp_gr.212373.116_Supplemental_Table_S1.docx]

Supplementary Table 1: Number of assayed positions in WE and WG simulations: This table lists the mean number of assayed positions, the number of informative (heterozygous) sites and the average distance between informative sites. Average distance between was calculated using sites on the p arm of chr1. All averages were calculated using 50 simulated samples per depth. There was a positive correlation between increasing depth and number of assayed sites, with a more pronounced effect in WE compared with WG. The interprobe distance is higher in the exome compared with the genome. This is due to having fewer sites and more variable distance between sites in WE compared with WG.

| **Depth (in x)** | **Platform** | **Mean # Assayed Positions** | **Mean # Informative Positions** | **Median Distance between Informative Positions** |
| --- | --- | --- | --- | --- |
| 20 | WG | 7,858,070 | 2,014,409 | 1,503 |
| 30 | WG | 7,866,967 | 1,949,467 | 1,554 |
| 40 | WG | 7,867,003 | 1,932,357 | 1,568 |
| 50 | WG | 7,867,003 | 1,924,407 | 1,574 |
| 50 | WE | 163,521 | 39,382 | 59,719 |
| 75 | WE | 181,053 | 43,131 | 54,581 |
| 100 | WE | 191,104 | 45,233 | 52,046 |
