# Supplementary material for: Detection of structural mosaicism from targeted and whole-genome sequencing data
Source: Genome Res. 2017 Oct;27(10):1704–14. doi: 10.1101/gr.212373.116 (PMC5630034; doi:10.1101/gr.212373.116)
Supplement: Supplemental Material [file supp_gr.212373.116_Supplemental_Table_S4.docx]

Supplementary Table 4: Rare variants in the most proximal region of the smallest LOH region: Despite the identification of a strong candidate gene in the region of interest and the interrogation of very high depth exome sequencing data, no loss-of-function or functional mutations were identified that were the obvious source of the reversion. Another possibility is that the suspected mutation responsible for driving the reversion may be absent from the exonic regions, i.e. is a regulatory mutation. PCR based amplification of this genomic region may be warranted and remains for future study.

| chr | pos | ref | alt | af | gene | ddg2p? | consequence |
| --- | --- | --- | --- | --- | --- | --- | --- |
| 11 | 92087959 | G | A | 0.005931 | FAT3 | no | missense_variant |
| 11 | 93170909 | T | TCC | none | CCDC67 | no | 3_prime_UTR_variant |
| 11 | 94039561 | G | A | 0.008177 | IZUMO1R | no | intron_variant |
| 11 | 94564757 | G | A | 0.000276 | AMOTL1 | no | intron_variant |
| 11 | 94696714 | T | C | 0.000366 | CWC15 | no | intron_variant |
| **11** | **95569170** | **T** | **G** | **0.007078** | **CEP57** | **yes** | **intron_variant** |
| 11 | 100665791 | C | T | 0.000414 | ARHGAP42_no | intron_variant | 11 |
