# Supplementary material for: Detection of structural mosaicism from targeted and whole-genome sequencing data
Source: Genome Res. 2017 Oct;27(10):1704–14. doi: 10.1101/gr.212373.116 (PMC5630034; doi:10.1101/gr.212373.116)
Supplement: Supplemental Material [file supp_gr.212373.116_Supplemental_Table_S2.docx]

Supplementary Table 2: Most mosaic copy number events escape detection by aCGH

| ID | tissue | chr | aCGH_appearance | clonality_by_SNP | detected_in_aCGH? |
| --- | --- | --- | --- | --- | --- |
| 265800 | blood* | 12 | no_deviation | absent | na |
| 265800 | saliva | 12 | no_data | 0.68 | na |
| 261373 | saliva | 12 | no_data | 0.45 | na |
| 261373 | blood* | 12 | no_deviation | absent | na |
| 273553 | blood* | 18 | no_deviation | absent | na |
| 273553 | saliva | 18 | no_data | 0.6 | na |
| 259003 | saliva* | 22 | deviation_but_no_call | 0.54 | no |
| 259003 | blood* | 22 | deviation_but_no_call | 0.34 | no |
| 274013 | blood* | 10 | no_deviation | absent | na |
| 274013 | saliva | 10 | no_data | 0.44 | na |
| 274600 | saliva | 18 | no_data | 0.49 | na |
| 274600 | blood* | 18 | no_deviation | absent | na |
| 260462 | saliva* | 18 | deviation_no_call | 0.5 | all-three-missed |
| 260462 | blood* | 18 | no_deviation | absent | na |
| 258956 | blood* | 3 | failed_QC | absent | na |
| 258956 | saliva* | 3 | partially_detected | 0.94 | yes |
| 261240 | blood* | 5 | no_data | absent | na |
| 261240 | saliva | 5 | partially_detected | 0.39 | partially_seen_escaped_review |
