# Supplementary material for: Detection of structural mosaicism from targeted and whole-genome sequencing data
Source: Genome Res. 2017 Oct;27(10):1704–14. doi: 10.1101/gr.212373.116 (PMC5630034; doi:10.1101/gr.212373.116)
Supplement: Supplemental Material [file supp_gr.212373.116_Supplemental_Table_S5.docx]

Supplementary Table 5: Rare variants in 257978 overlapping mosaic UPD region: Investigation of rare variants overlapping the mosaic LOH interval identified no loss of function mutations in this region.

| chr | pos | ref | alt | af | gene | ddg2p? | consequence |
| --- | --- | --- | --- | --- | --- | --- | --- |
| 5 | 102891545 | ACT | A | 0.002929 | NUDT12 | no | intron_variant&feature_truncation |
| 5 | 106532701 | G | C | 0.000122 | PSMC1P5 | no | downstream_gene_variant |
| 5 | 110560413 | C | T | 0.008055 | CAMK4 | no | intron_variant |
| 5 | 110814291 | AC | A | 0.008787 | CAMK4 | no | intron_variant&feature_truncation |
| 5 | 110814299 | TAA | T | 0.008787 | CAMK4 | no | intron_variant&feature_truncation |
| 5 | 111541024 | C | CAGCCA | 0.002621 | EPB41L4A | no | intron_variant&feature_elongation |
| 5 | 122152521 | G | C | 0.00331 | SNX2 | no | intron_variant |
| 5 | 127610125 | C | T | 0.000488 | FBN2 | yes | intron_variant |
| 5 | 133644012 | A | G | 0.003417 | CDKL3 | no | missense_variant |
| 5 | 134910284 | T | C | 0.000244 | CXCL14 | no | missense_variant |
| 5 | 140752319 | T | C | 0.000244 | PCDHGA1&PCDHGA2&PCDHGA3&PCDHGA4&PCDHGA5&PCDHGA6&PCDHGB1&PCDHGB2&PCDHGB3 | no | synonymous_variant |
| 5 | 140908588 | G | C | 0.000976 | DIAPH1 | no | intron_variant |
| 5 | 141033714 | ACCCCCGTGCCTG | A | 0.004882 | ARAP3&FCHSD1 | no | inframe_deletion |
| 5 | 147473877 | A | G | 0.000138 | SPINK5 | no | intron_variant |
| 5 | 147516521 | C | G | 0.002069 | SPINK5 | no | intron_variant |
| 5 | 159766366 | CAAAA | CAAAAAA | 0.007689 | CCNJL | no | intron_variant&nc_transcript_variant&feature_elongation |
| 5 | 162868819 | A | G | 0.003417 | CCNG1&NUDCD2 | no | intron_variant |
| 5 | 169097441 | A | G | 0.008909 | DOCK2 | no | intron_variant |
| 5 | 170336582 | CTTTTT | CTTTTTT | 0.009519 | RANBP17 | no | intron_variant&feature_elongation |
| 5 | 175819928 | G | A | 0.009275 | ARL10&CLTB&HIGD2A&NOP16 | no | missense_variant |
| 5 | 175915856 | G | C | 0.000366 | FAF2 | no | intron_variant |
| 5 | 177570597 | G | C | 0.001098 | RMND5B | no | intron_variant |
| 5 | 180219193 | C | T | 0.000122 | MGAT1 | no | missense_variant |
